# Association Between Serum Parathyroid Hormone Levels and Femoral Bone Mineral Density in Patients with Chronic Kidney Disease Stage 3

**DOI:** 10.3390/jcm15145519

**Published:** 2026-07-14

**Authors:** Laura Montaño-Azor, Petra Cantón Guerrero, María Ángeles Jiménez Sánchez, María José Jiménez Moral, Raquel María García-Sáez, María Encarnación Rodríguez-Ortiz, Mariano Rodríguez, Victoria Pendón-Ruiz de Mier, Esperanza Romero-Rodríguez

**Affiliations:** 1 Multiprofessional Teaching Unit for Family and Community Care of Córdoba, 14011 Cordoba, Spain; lauramonazor@gmail.com; 2 Palma del Río Health Center, Andalusian Health Service, 14700 Cordoba, Spain; 3 Córdoba Guadalquivir Health District, Andalusian Health Service, 14011 Cordoba, Spainemromerorodriguez@gmail.com (E.R.-R.); 4 Maimonides Institute for Biomedical Research of Cordoba (IMIBIC), 14004 Cordoba, Spain; mariajose.jimenez@imibic.org (M.J.J.M.); maria.rodriguez@imibic.org (M.E.R.-O.); mvictoriaprm@gmail.com (V.P.-R.d.M.); 5 Nephrology Service, Reina Sofía University Hospital, 14004 Córdoba, Spain; 6 University of Cordoba, 14004 Cordoba, Spain; 7 Redes de Investigación Cooperativa Orientadas a Resultados en Salud (RICORS), Carlos III Health Institute, 28029 Madrid, Spain; 8 Carlos Castilla del Pino Health Center, Andalusian Health Service, 14011 Cordoba, Spain

**Keywords:** chronic kidney disease, parathyroid hormone, bone mineral density, chronic kidney disease–mineral and bone disorder, fibroblast growth factor 23, secondary hyperparathyroidism, dual-energy X-ray absorptiometry

## Abstract

**Background**: Chronic kidney disease (CKD) is associated with early disturbances in mineral metabolism that contribute to bone fragility. Secondary hyperparathyroidism is a key component of chronic kidney disease–mineral and bone disorder (CKD-MBD) and may affect bone even during the early stages of CKD. This study evaluated the association between serum parathyroid hormone (PTH) concentrations and bone mineral density (BMD) in patients with stage 3 CKD. **Methods**: A multicenter cross-sectional observational study was conducted in 203 patients with stage 3 CKD. Blood and 24 h urine samples were collected to measure creatinine, calcium, phosphate, magnesium, 25-hydroxyvitamin D, fibroblast growth factor 23 (FGF23), and PTH. Bone mineral density was assessed by dual-energy X-ray absorptiometry (DXA). Associations between serum PTH concentrations and BMD were analyzed. **Results**: Elevated serum PTH concentrations were associated with lower femoral BMD and less favorable femoral T-scores, whereas no significant associations were observed at the lumbar spine. Serum PTH concentrations were inversely correlated with femoral BMD and femoral T-scores. Patients with reduced femoral BMD also showed higher FGF23 concentrations and lower renal function, while serum calcium, phosphate, magnesium, and 25-hydroxyvitamin D concentrations did not differ significantly among the groups. **Conclusions**: Elevated serum PTH concentrations were associated with reduced femoral BMD in patients with stage 3 CKD, supporting preferential early involvement of cortical bone. Serum PTH may represent an accessible biomarker for the early identification of patients at increased risk of cortical bone loss before conventional biochemical abnormalities become clinically apparent.

## 1. Introduction

Chronic kidney disease (CKD) is characterized by a progressive decline in renal function and profound alterations in mineral metabolism. It is defined by the presence of kidney damage or a glomerular filtration rate (GFR) below 60 mL/min/1.73 m^2^ for at least three months, with important implications for skeletal and cardiovascular health [1,2]. One of the earliest metabolic disturbances in CKD is the disruption of calcium–phosphate homeostasis, which triggers compensatory endocrine responses aimed at maintaining mineral balance [3,4,5,6].

The parathyroid gland plays a fundamental role in mineral metabolism through the secretion of parathyroid hormone (PTH) [7,8,9]. PTH acts on the kidney by increasing tubular calcium reabsorption, reducing phosphate reabsorption (thereby promoting phosphaturia), and stimulating renal 1α-hydroxylase activity, which increases calcitriol synthesis. Calcitriol, in turn, enhances intestinal calcium and phosphate absorption. In bone, sustained PTH exposure increases osteoclastic bone resorption through the indirect activation of osteoclastogenesis via osteoblast-derived receptor activator of nuclear factor kappa-B ligand (RANKL), contributing to accelerated bone remodeling, particularly in cortical bone, with progressive cortical porosity and bone loss.

As renal function declines, phosphate retention and reduced calcitriol synthesis stimulate a progressive increase in PTH secretion, leading to secondary hyperparathyroidism [3,4,5,6,10,11,12,13]. In parallel, fibroblast growth factor 23 (FGF23), a phosphaturic hormone secreted by osteocytes and osteoblasts, increases early in CKD as a compensatory mechanism to enhance urinary phosphate excretion and suppress calcitriol synthesis. Although initially adaptive, persistent elevation of FGF23 contributes to the complex endocrine dysregulation characteristic of CKD-MBD through its interactions with vitamin D and PTH, ultimately disrupting mineral homeostasis [14,15,16,17,18]. Together, these endocrine alterations have been associated with bone loss, fractures, cardiovascular disease, and increased mortality in patients with CKD [19,20,21,22].

Bone mineral density (BMD), assessed by dual-energy X-ray absorptiometry (DXA), is widely used to evaluate bone health and fracture risk. Previous studies suggest that elevated PTH preferentially affects cortical bone, such as the femur, whereas trabecular bone, predominantly represented by the lumbar spine, appears relatively preserved during the early stages of CKD. This site-specific pattern has been attributed to the higher remodeling rate and increased cortical porosity induced by sustained PTH exposure, although the underlying mechanisms remain incompletely understood [12,23,24,25,26].

Although CKD-MBD has traditionally been associated with advanced CKD, increasing evidence indicates that abnormalities in bone remodeling begin much earlier, even before overt disturbances in serum calcium and phosphate become evident [3,4,5,6]. Mild elevations in serum PTH may therefore represent one of the earliest adaptive responses to declining renal function while simultaneously contributing to preferential cortical bone loss. Importantly, normal serum calcium and phosphate concentrations do not necessarily exclude ongoing skeletal deterioration, as compensatory hormonal mechanisms involving PTH and FGF23 maintain serum mineral homeostasis despite progressive alterations in bone remodeling [3,4,5,6,10,11,12,14].

Despite these advances, the relationship between serum PTH concentrations and site-specific bone mineral density in patients with stage 3 CKD remains incompletely understood. Most previous studies have focused on advanced CKD or dialysis-dependent populations, whereas the early skeletal changes that occur before overt biochemical abnormalities and established CKD-MBD have been less extensively investigated. A better understanding of these early alterations could improve the identification of patients at increased risk of cortical bone loss and facilitate earlier preventive interventions. Therefore, evaluating the potential role of serum PTH as a readily available biomarker of early skeletal involvement may have important clinical implications for risk stratification and the timely implementation of strategies aimed at preserving bone health [3,4,5,6,10,11,12,14,20,26,27].

Therefore, the aim of this study was to evaluate the association between serum PTH concentrations and bone mineral density at the lumbar spine and femoral sites in patients with stage 3 CKD. We also evaluated whether serum PTH may represent an early marker of preferential cortical bone involvement before conventional biochemical abnormalities become clinically evident.

## 2. Materials and Methods

### 2.1. Study Design

This multicenter, observational, descriptive study was conducted in the province of Córdoba, Spain, by the Córdoba–Guadalquivir Primary Care Health District in collaboration with Reina Sofía University Hospital and the Maimonides Institute for Biomedical Research of Córdoba (IMIBIC). The study was conducted in accordance with the Declaration of Helsinki (1975, revised in 2013) and was approved by the Research Ethics Committee of Córdoba (registration No. 330; reference No. 5276). All participants provided written informed consent prior to enrollment. The study is reported in accordance with the Strengthening the Reporting of Observational Studies in Epidemiology (STROBE) Statement [28].

Participants were recruited consecutively between January 2022 and June 2024 from primary care centers within the Córdoba–Guadalquivir Health District.

A total of 203 patients were included in the study. Descriptive analyses were performed using the entire study population. For each specific analysis, all participants with complete data for the variables of interest were included. Consequently, sample sizes differed slightly across analyses because of missing laboratory or densitometric measurements, incomplete records, or unavailable measurements rather than patient exclusion. The number of participants included in each analysis is indicated in the corresponding tables.

Patients fulfilling all inclusion criteria and none of the exclusion criteria were eligible for participation. Eligible participants were adults (≥18 years) with stage 3 CKD. CKD stage 3 was defined by an estimated glomerular filtration rate (eGFR), calculated using the Chronic Kidney Disease Epidemiology Collaboration (CKD-EPI) creatinine equation [29], between 30 and 60 mL/min/1.73 m^2^ for at least three months. Women of childbearing potential were required to have a negative pregnancy test before enrollment. Participants had to be able to understand the study procedures and provide written informed consent.

Patients with specific renal diseases, including glomerulonephritis, polycystic kidney disease, obstructive nephropathy, nephrolithiasis, or tubulointerstitial nephropathy, were excluded. Additional exclusion criteria included proteinuria greater than 300 mg/24 h, hyperphosphatemia (serum phosphate > 4.5 mg/dL), hypermagnesemia (serum magnesium > 2.6 mg/dL), active infection within the previous 30 days, systemic inflammatory disease, infection with human immunodeficiency virus or hepatitis B or C virus, chronic liver disease, or a history of malignancy within the previous five years.

Patients receiving treatment with bisphosphonates, denosumab, biologic therapies for autoimmune diseases, or immunosuppressive drugs were also excluded. Pregnant or breastfeeding women, women planning pregnancy, individuals with active drug or alcohol abuse that could interfere with study procedures, patients participating in other clinical trials, and those unable or unwilling to provide written informed consent were also excluded.

### 2.2. Laboratory Analysis and Calculation of Study Parameters

The following biochemical and urinary variables related to chronic kidney disease–mineral and bone disorder (CKD-MBD) and renal function were analyzed: serum calcium, phosphate, magnesium, fibroblast growth factor 23 (FGF23), 25-hydroxyvitamin D, parathyroid hormone (PTH), creatinine, and estimated glomerular filtration rate (eGFR). These biomarkers were selected because of their established roles in mineral metabolism, secondary hyperparathyroidism, and bone remodeling in patients with CKD. Urinary parameters included 24 h urinary excretion of calcium, phosphate, protein, and creatinine, as well as derived indices reflecting renal tubular handling of minerals.

Biochemical determinations were performed using an ADVIA Chemistry analyzer (Siemens Healthcare Diagnostics, Erlangen, Germany). Intact FGF23 (iFGF23) was measured by enzyme-linked immunosorbent assay (ELISA; Kainos Laboratories, Tokyo, Japan), which specifically detects the biologically active intact protein. Serum PTH concentrations were determined by immunoradiometric assay (IRMA; N-Tact^®^ PTH SP IRMA, DiaSorin S.p.A., Saluggia, Italy). Twenty-four-hour urine samples were collected on the day before blood sampling. Urinary phosphate, calcium, protein, and creatinine concentrations were measured using an Architect c16000 analyzer (Abbott^®^, Chicago, IL, USA).

Estimated glomerular filtration rate (eGFR) was calculated using the Chronic Kidney Disease Epidemiology Collaboration (CKD-EPI) creatinine equation [29].

Daily urinary phosphate and calcium excretion were expressed as total 24 h urinary excretion and additionally normalized to eGFR (mg/24 h per mL/min/1.73 m^2^) to provide an indirect estimate of mineral load adjusted for renal function. Urinary calcium excretion was expressed as the calcium-to-creatinine ratio, whereas renal phosphate handling was assessed by the fractional excretion of phosphate (FEP), calculated as:

$$FEP\left(\%\right)=\frac{Urinary\;phosphate\times Serum\;creatinine}{Serum\;phosphate\times Urinary\;creatinine}\times 100$$

For analytical purposes, serum PTH concentrations were categorized according to the reference range of the assay. Patients were classified using the upper limit of normality (88 pg/mL) as the predefined cutoff. This cutoff corresponded to the upper limit of the reference range established by our institutional laboratory and was selected to identify patients with biochemical evidence of secondary hyperparathyroidism. Normal PTH concentrations were defined as 18.5–88 pg/mL, whereas elevated PTH concentrations were defined as >88 pg/mL.

Bone mineral density (BMD) was assessed by dual-energy X-ray absorptiometry (DXA) at the lumbar spine (L1–L4) and hip according to standard clinical practice and classified according to the World Health Organization (WHO) criteria [30] based on T-scores. Normal BMD was defined as a T-score ≥ −1.0, osteopenia as a T-score between −1.0 and −2.5, and osteoporosis as a T-score ≤ −2.5.

### 2.3. Statistical Analysis

Statistical analyses were performed using SPSS software (version 25.0; IBM Corp., Armonk, NY, USA). All statistical tests were two-sided, and a *p* value < 0.05 was considered statistically significant.

A descriptive analysis was first performed. Categorical variables are presented as absolute and relative frequencies, whereas continuous variables are expressed as mean ± standard deviation (SD). Ninety-five percent confidence intervals (95% CI) were calculated when appropriate.

The distribution of continuous variables was assessed before selecting the appropriate statistical tests. Comparisons between two independent groups were performed using Student’s t-test for normally distributed variables or the Mann–Whitney U test for non-normally distributed variables. Comparisons among three or more groups were performed using one-way analysis of variance (ANOVA) with Scheffé’s post hoc test or the Kruskal–Wallis test, as appropriate.

Categorical variables were compared using the chi-square test or Fisher’s exact test when expected cell frequencies were <5.

Correlation analyses were performed to evaluate the relationships between serum PTH concentrations, bone mineral density, and variables related to mineral metabolism and renal function.

For each analysis, only participants with complete data for the variables of interest were included. No imputation of missing data was performed. Consequently, sample sizes varied slightly across analyses according to the availability of complete laboratory and densitometric measurements.

## 3. Results

### 3.1. Baseline Clinical and Biochemical Characteristics

Baseline clinical and biochemical characteristics of the study population, stratified by sex, are presented in Table 1. A total of 203 patients with stage 3 chronic kidney disease (CKD) were included in the study. The mean age was 68.8 ± 9.8 years, 67.0% of participants were women, mean estimated glomerular filtration rate (eGFR) was 53.8 ± 12.4 mL/min/1.73 m^2^, and mean serum parathyroid hormone (PTH) concentration was 85.0 ± 64.0 pg/mL.

Women were significantly older than men (70.0 ± 9.6 vs. 66.4 ± 9.9 years, *p* = 0.015), whereas men had significantly higher serum creatinine concentrations (1.46 ± 0.33 vs. 1.13 ± 0.25 mg/dL, *p* < 0.001). Women also had significantly higher urinary calcium-to-creatinine and phosphate-to-creatinine ratios than men (*p* = 0.002 and *p* < 0.001, respectively).

Bone mineralparameters also differed according to sex. Men showed higher lumbar spine and femoral BMD values, together with more favorable lumbar spine and femoral T-scores, compared with women (all *p* < 0.05).

No statistically significant differences were observed between men and women in body mass index, eGFR, serum calcium, phosphate, magnesium, FGF23, 25-hydroxyvitamin D, PTH concentrations, urinary protein-to-creatinine ratio, or fractional excretion of phosphate (all *p* > 0.05).

### 3.2. Comparison According to Serum PTH Levels

Patients were classified into normal and elevated PTH groups according to the predefined laboratory cutoff values (normal, 18.5–88 pg/mL; elevated, >88 pg/mL). Serum PTH measurements were available for 185 participants, who were included in this analysis.

As shown in Table 2, patients with elevated PTH concentrations were significantly older than those with normal PTH concentrations (72.12 ± 7.50 vs. 67.35 ± 10.19 years, *p* = 0.004) and had significantly lower estimated glomerular filtration rate (eGFR) (48.76 ± 10.90 vs. 58.07 ± 12.29 mL/min/1.73 m^2^, *p* < 0.001). No significant differences were observed between groups in body mass index, serum creatinine, calcium, phosphate, magnesium, 25-hydroxyvitamin D, FGF23, or urinary protein-to-creatinine ratio (all *p* > 0.05).

Regarding urinary mineral handling, patients with elevated PTH concentrations exhibited significantly higher fractional excretion of phosphate (FEP) (*p* = 0.030), together with significantly lower urinary calcium-to-creatinine (*p* = 0.002) and urinary phosphate-to-creatinine ratios (*p* < 0.001), compared with patients with normal PTH concentrations.

Regarding skeletal parameters, patients with elevated PTH concentrations showed significantly lower femoral bone mineral density (BMD) and femoral T-scores than those with normal PTH concentrations (both *p* < 0.001). In contrast, lumbar spine BMD and lumbar spine T-scores did not differ significantly between the two groups.

Continuous variables are presented as mean ± SD unless otherwise indicated. Normal PTH: 18.5–88 pg/mL; Elevated PTH: >88 pg/mL. Only participants with available serum PTH measurements were included in this analysis (*n* = 185).

### 3.3. Analysis According to Femoral Bone Mineral Density

Bone mineral density data were available for 188 of the 203 participants and were used for the analysis according to femoral BMD categories.

As shown in Table 3, age differed significantly across femoral BMD categories (*p* < 0.001), with progressively higher values in patients with osteopenia and osteoporosis. Renal function progressively declined across femoral BMD categories, as reflected by lower estimated glomerular filtration rate (eGFR) (*p* = 0.005) and higher serum creatinine concentrations (*p* = 0.043).

Among the biomarkers of mineral metabolism, serum PTH concentrations increased progressively from the normal BMD group to the osteoporosis group (*p* < 0.001). Serum FGF23 concentrations also differed significantly across femoral BMD categories (*p* = 0.010). In contrast, no significant differences were observed in serum calcium, phosphate, magnesium, or 25-hydroxyvitamin D concentrations (all *p* > 0.05).

No statistically significant differences were observed among femoral BMD categories in urinary protein-to-creatinine ratio, urinary calcium-to-creatinine ratio, urinary phosphate-to-creatinine ratio, or fractional excretion of phosphate (all *p* > 0.05).

Femoral BMD and femoral T-scores progressively decreased across the three categories (both *p* < 0.001). Lumbar spine BMD and lumbar spine T-scores also differed significantly among the three groups (both *p* < 0.001).

### 3.4. Correlation Analyses

Correlation analyses were performed to evaluate the relationships between serum PTH concentrations, bone mineral density, and variables related to mineral metabolism and renal function. The correlation coefficients are summarized in Table 4.

Serum PTH concentrations were inversely correlated with femoral bone mineral density (BMD) (r = −0.36, *p* < 0.001) and femoral T-score (r = −0.35, *p* < 0.001). In contrast, no significant correlations were observed between serum PTH concentrations and lumbar spine BMD (r = −0.12, *p* = 0.37) or lumbar spine T-score (r = −0.11, *p* = 0.37).

**Figure 1:**
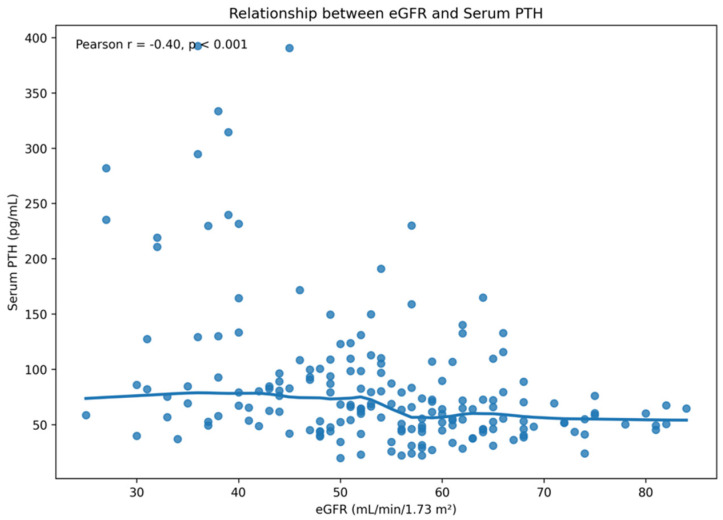
Relationship between serum parathyroid hormone (PTH) concentrations and estimated glomerular filtration rate (eGFR). Serum PTH concentrations increased as renal function declined. Pearson correlation analysis demonstrated a moderate inverse correlation between serum PTH and eGFR (r = −0.41, *p* < 0.001). The fitted smoothing curve suggests a steeper increase in PTH concentrations at eGFR values below approximately 40 mL/min/1.73 m^2^.

Serum PTH concentrations were positively correlated with the fractional excretion of phosphate (FEP) (r = 0.29, *p* < 0.001). In addition, serum PTH concentrations were inversely correlated with estimated glomerular filtration rate (eGFR), with higher PTH concentrations observed as renal function declined. As illustrated in Figure 1, Pearson correlation analysis demonstrated a moderate inverse correlation between serum PTH concentrations and eGFR (r = −0.41, *p* < 0.001).

No significant correlations were observed between serum PTH concentrations and serum calcium, phosphate, magnesium, or 25-hydroxyvitamin D concentrations (all *p* > 0.05).

## 4. Discussion

In this multicenter study of patients with stage 3 chronic kidney disease (CKD), elevated serum parathyroid hormone (PTH) concentrations were significantly associated with lower femoral bone mineral density (BMD) and less favorable femoral T-scores, whereas no significant association was observed at the lumbar spine. These findings are consistent with preferential early involvement of cortical bone during the initial stages of CKD–mineral and bone disorder (CKD-MBD). In addition, higher serum PTH concentrations were associated with declining renal function and altered phosphate handling, supporting the close interaction between kidney dysfunction, secondary hyperparathyroidism, and early skeletal changes.

Our finding that elevated serum PTH concentrations were associated with femoral but not lumbar BMD is consistent with previous studies showing that secondary hyperparathyroidism predominantly affects cortical rather than trabecular bone [3,4,12,25]. This site-specific pattern is biologically plausible because chronic PTH excess preferentially stimulates intracortical remodeling. Activation of the PTH1 receptor in osteoblasts increases receptor activator of nuclear factor kappa-B ligand (RANKL) expression while suppressing osteoprotegerin production, thereby promoting osteoclast differentiation and bone resorption. As a consequence, cortical bone develops increased porosity and progressive thinning, whereas trabecular bone is relatively preserved during the early stages of CKD [12,25]. This differential response may reflect the greater surface area available for intracortical remodeling and the higher susceptibility of cortical bone to sustained PTH exposure [12,25].

An additional explanation for the absence of significant associations at the lumbar spine relates to the intrinsic limitations of dual-energy X-ray absorptiometry (DXA) in this skeletal region. Degenerative changes, osteophytes, vertebral sclerosis, and vascular calcifications frequently present in older individuals and patients with CKD may artificially increase lumbar BMD measurements, potentially masking early trabecular bone loss [26]. Consequently, femoral BMD may represent a more sensitive marker of early skeletal involvement in patients with stage 3 CKD.

The observed increase in PTH concentrations should be interpreted within the complex endocrine adaptations that characterize early CKD-MBD. Declining renal function promotes phosphate retention and reduces renal expression of α-Klotho, leading to an early increase in FGF23 secretion by osteocytes. Elevated FGF23 enhances urinary phosphate excretion while simultaneously suppressing renal 1α-hydroxylase activity, resulting in reduced calcitriol synthesis. Lower calcitriol concentrations decrease intestinal calcium absorption, providing an important stimulus for compensatory PTH secretion [14,15,16,17,18].

Importantly, these endocrine adaptations explain why serum calcium and phosphate concentrations frequently remain within the normal range during the early stages of CKD despite ongoing skeletal remodeling. Increased PTH secretion together with elevated FGF23 maintains phosphate balance by enhancing phosphaturia and preserves serum calcium through increased bone resorption and renal calcium reabsorption. Consequently, normal biochemical parameters do not necessarily exclude active bone disease, a concept emphasized in the current KDIGO recommendations [3,20].

Although FGF23 was not the primary focus of the present study, the higher circulating FGF23 concentrations observed in patients with reduced femoral BMD further support the concept that endocrine disturbances begin early during CKD progression. Previous studies have demonstrated that FGF23 rises before overt hyperphosphatemia and frequently precedes marked elevations in serum PTH [14,15,16,17,18]. Our findings are consistent with this model and suggest that the coexistence of elevated FGF23 and PTH may identify patients with early CKD-MBD despite preserved conventional biochemical markers. These findings are in agreement with current models of CKD-MBD, in which endocrine adaptations precede measurable biochemical abnormalities and structural skeletal damage becomes detectable before overt disturbances in serum calcium or phosphate concentrations [3,14,20].

The present study extends previous observations by demonstrating that the association between elevated serum PTH concentrations and reduced bone mineral density is already evident in patients with stage 3 CKD, before the development of overt biochemical abnormalities typically associated with advanced CKD-MBD. Unlike many previous studies performed in patients with advanced CKD or dialysis populations, our cohort represents an earlier stage of kidney disease in which conventional biochemical markers such as serum calcium and phosphate remained largely within the normal range. These findings support the concept that serum PTH may serve as an early and readily available biomarker of cortical bone involvement in CKD [3,4,12,20,26].

From a clinical perspective, these findings suggest that monitoring serum PTH concentrations together with femoral DXA assessment may improve the early identification of patients at increased risk of skeletal deterioration. Since lumbar spine measurements may underestimate early cortical bone loss, preferential assessment of femoral BMD could provide additional clinical value in patients with stage 3 CKD. Earlier recognition of CKD-MBD may facilitate timely preventive interventions before clinically overt bone disease or fractures develop.

This study has several limitations that should be acknowledged. First, its cross-sectional design precludes establishing causal relationships between serum PTH concentrations and bone mineral density. Second, the relatively small number of patients with osteoporosis may have limited statistical power for subgroup analyses. Third, bone quality and turnover were not directly assessed because bone histomorphometry was not performed, and DXA cannot fully characterize bone microarchitecture in patients with CKD. Finally, residual confounding related to factors such as dietary intake, physical activity, medication use, diabetes, obesity, and age cannot be completely excluded. Although age, obesity, and diabetes may have influenced bone mineral density, these variables were not the primary focus of the present study and therefore should be considered when interpreting the findings.

Despite these limitations, this study has several strengths, including the multicenter design, the comprehensive assessment of biomarkers related to CKD-MBD, and the simultaneous evaluation of serum PTH, FGF23, vitamin D, renal function, and site-specific bone mineral density in patients with early-stage CKD. To our knowledge, few previous studies have simultaneously evaluated these parameters in a multicenter cohort of patients with stage 3 CKD. This comprehensive evaluation provides novel evidence supporting the association between early PTH elevation and preferential cortical bone loss in patients with stage 3 CKD.

Future prospective longitudinal studies are warranted to determine whether elevated serum PTH concentrations predict subsequent bone loss, fractures, and adverse clinical outcomes, and to establish whether early identification of patients with elevated PTH may improve long-term skeletal outcomes in CKD.

Overall, our findings support the potential role of serum PTH as an early biomarker of cortical bone involvement in patients with stage 3 CKD and reinforce the importance of early assessment of CKD-MBD before conventional biochemical abnormalities become clinically apparent.

## 5. Conclusions

Elevated serum parathyroid hormone (PTH) concentrations were associated with lower femoral bone mineral density (BMD) and less favorable femoral T-scores in patients with stage 3 chronic kidney disease (CKD), whereas no significant associations were observed at the lumbar spine. These findings are consistent with preferential early involvement of cortical bone during the initial stages of chronic kidney disease–mineral and bone disorder (CKD-MBD).

Although the cross-sectional design precludes causal inference, the observed associations suggest that disturbances in mineral metabolism and skeletal involvement may occur before overt abnormalities in conventional biochemical markers, such as serum calcium and phosphate, become clinically apparent.

Our findings support the potential role of serum PTH as an accessible biomarker for the early identification of patients at increased risk of cortical bone loss. The combined assessment of serum PTH and femoral DXA may improve the early recognition of CKD-related bone disease and facilitate timely preventive strategies. Prospective longitudinal studies are warranted to confirm these findings and determine whether early identification of elevated PTH improves the prediction of subsequent bone loss and fracture risk.

## Figures and Tables

**Table 1:** Baseline clinical and biochemical characteristics of patients with chronic kidney disease (CKD) stage 3, stratified by sex. Abbreviations: BMI, body mass index; eGFR, estimated glomerular filtration rate; CKD-EPI, Chronic Kidney Disease Epidemiology Collaboration; FGF23, fibroblast growth factor; PTH, parathyroid hormone; FEP, Fractional excretion of phosphate.

Variable	Total (*n* = 203)	Men (*n* = 67)	Women (*n* = 136) *p*-Value
Patients, n (%)	203 (100%)	67 (33.0%)	136 (67.0%)
Age (years)	68.83 ± 9.82	66.42 ± 9.91	70.00 ± 9.57 0.015
BMI (kg/m^2^)	30.17 ± 5.13	29.63 ± 4.89	30.43 ± 5.25 0.282
eGFR, CKD-EPI (mL/min/1.73 m^2^)	53.75 ± 12.44	55.94 ± 12.73	52.66 ± 12.23 0.083
Creatinine (mg/dL)	1.24 ± 0.32	1.46 ± 0.33	1.13 ± 0.25 <0.001
Calcium (mg/dL)	9.67 ± 0.36	9.70 ± 0.34	9.66 ± 0.37 0.356
Phosphate (mg/dL)	3.41 ± 0.57	3.49 ± 0.56	3.37 ± 0.57 0.152
Magnesium (mg/dL)	1.99 ± 0.27	1.94 ± 0.28	2.01 ± 0.26 0.163
FGF23 (pg/mL)	89.54 ± 93.96	77.86 ± 45.94	95.00 ± 109.29 0.550
25-hydroxyvitamin D (ng/mL)	17.98 ± 12.03	17.12 ± 8.47	18.44 ± 13.58 0.842
PTH (pg/mL)	85.00 ± 64.00	79.46 ± 57.98	87.77 ± 66.78 0.349
Urinary protein/creatinine (mg/g)	287.81 ± 509.46 (*n* = 102)	397.96 ± 749.05 (*n* = 42)	210.70 ± 199.34 (*n* = 60) 0.430
Urinary calcium/creatinine (mg/g)	87.60 ± 63.87	68.69 ± 53.65	96.98 ± 66.61 0.002
Urinary phosphate/creatinine (mg/g)	675.38 ± 194.61	600.11 ± 156.31	712.71 ± 201.37 <0.001
FEP (%)	24.89 ± 11.47	26.91 ± 12.02	23.89 ± 11.08 0.083
Lumbar spine BMD (g/cm^2^)	1.15 ± 0.19 (*n* = 194)	1.25 ± 0.21 (*n* = 63)	1.10 ± 0.17 (*n* = 131) <0.001
Lumbar spine T-score	−0.36 ± 1.57 (*n* = 194)	0.25 ± 1.77 (*n* = 63)	−0.65 ± 1.37 (*n* = 131) 0.001
Femoral BMD (g/cm^2^)	0.94 ± 0.16 (*n* = 188)	1.02 ± 0.16 (*n* = 64)	0.89 ± 0.14 (*n* = 124) <0.001
Femoral T-score	−0.75 ± 1.20 (*n* = 188)	−0.46 ± 1.24 (*n* = 64)	−0.90 ± 1.16 (*n* = 124) 0.018

Abbreviations: BMI, body mass index; eGFR, estimated glomerular filtration rate; CKD-EPI, Chronic Kidney Disease Epidemiology Collaboration; FGF23, fibroblast growth factor 23; PTH, parathyroid hormone; FEP, fractional excretion of phosphate. Data are presented as mean ± SD unless otherwise indicated. Values of n shown for specific variables indicate the number of participants with available data because of unavailable laboratory or densitometric measurements.

**Table 2:** Comparison of clinical variables and bone mineral density according to serum parathyroid hormone levels. Abbreviations: BMI, body mass index; eGFR, estimated glomerular filtration rate; CKD-EPI, Chronic Kidney Disease Epidemiology Collaboration; FGF23, fibroblast growth factor; BMD, bone mineral density; FEP, Fractional excretion of phosphate.

Variable	Normal PTH (*n* = 132)	Elevated PTH (*n* = 53)	*p*-Value
Age (years)	67.35 ± 10.19	72.12 ± 7.50	0.004
BMI (kg/m^2^)	30.58 ± 5.61	29.99 ± 5.55	0.589
eGFR, CKD-EPI (mL/min/1.73 m^2^)	58.07 ± 12.29	48.76 ± 10.90	<0.001
Creatinine (mg/dL)	1.22 ± 0.31	1.30 ± 0.32	0.08
Calcium (mg/dL)	9.70 ± 0.38	9.60 ± 0.64	0.358
Phosphate (mg/dL)	3.48 ± 0.49	3.51 ± 0.69	0.782
Magnesium (mg/dL)	2.00 ± 0.27	1.96 ± 0.26	0.392
25-hydroxyvitamin D (ng/mL)	18.44 ± 13.58	17.12 ± 8.47	0.842
FGF23 (pg/mL)	82.31 ± 71.42	106.43 ± 129.67	0.365
Urinary protein/creatinine (mg/g)	257.91 ± 424.87	351.62 ± 642.18	0.430
Urinary calcium/creatinine (mg/g)	96.98 ± 66.61	68.69 ± 53.65	0.002
Urinary phosphate/creatinine (mg/g)	712.71 ± 201.37	600.11 ± 156.31	<0.001
FEP (%)	23.89 ± 11.08	26.91 ± 12.02	0.030
Lumbar spine BMD (g/cm^2^)	1.18 ± 0.19	1.14 ± 0.20	0.370
Lumbar spine T-score	−0.11 ± 1.47	−0.38 ± 1.60	0.373
Femoral BMD (g/cm^2^)	0.97 ± 0.17	0.86 ± 0.12	<0.001
Femoral T-score	−0.50 ± 1.30	−1.32 ± 0.99	<0.001

Abbreviations: BMI, body mass index; eGFR, estimated glomerular filtration rate; CKD-EPI, Chronic Kidney Disease Epidemiology Collaboration; FGF23, fibroblast growth factor 23; BMD, bone mineral density; FEP, fractional excretion of phosphate.

**Table 3:** Comparison of clinical and biochemical variables according to femoral bone mineral density (BMD). Abbreviations: BMI, body mass index; eGFR, estimated glomerular filtration rate; CKD-EPI, Chronic Kidney Disease Epidemiology Collaboration; FGF23, fibroblast growth factor; PTH, parathyroid hormone; BMD, bone mineral density; FEP, Fractional excretion of phosphate.

Variable	Normal BMD (*n* = 108)	Osteopenia (*n* = 69)	Osteoporosis (*n* = 11)	*p*-Value
Age (years)	65.93 ± 10.77	72.42 ± 6.37	77.57 ± 5.80	<0.001
BMI (kg/m^2^)	30.82 ± 4.66	29.29 ± 5.37	28.14 ± 4.30	0.078
eGFR, CKD-EPI (mL/min/1.73 m^2^)	55.26 ± 12.60	52.46 ± 12.16	42.91 ± 9.27	0.005
Creatinine (mg/dL)	1.24 ± 0.31	1.23 ± 0.33	1.41 ± 0.24	0.043
Calcium (mg/dL)	9.71 ± 0.39	9.72 ± 0.55	9.70 ± 0.40	0.938
Phosphate (mg/dL)	3.41 ± 0.53	3.57 ± 0.55	3.29 ± 0.67	0.166
Magnesium (mg/dL)	1.98 ± 0.29	1.99 ± 0.26	2.11 ± 0.30	0.285
25-hydroxyvitamin D (ng/mL)	18.09 ± 12.28	18.38 ± 12.55	16.22 ± 10.48	0.893
FGF23 (pg/mL)	68.07 ± 22.98	120.30 ± 146.02	93.04 ± 31.74	0.010
PTH (pg/mL)	67.86 ± 34.01	102.77 ± 77.67	177.07 ± 112.71	<0.001
Urinary protein/creatinine (mg/g)	274.94 ± 625.81	323.07 ± 333.05	377.75 ± 534.22	0.139
Urinary calcium/creatinine (mg/g)	88.26 ± 60.53	86.78 ± 64.00	68.10 ± 68.23	0.319
Urinary phosphate/creatinine (mg/g)	654.58 ± 192.14	710.69 ± 184.29	668.50 ± 228.50	0.184
FEP (%)	23.84 ± 8.67	26.13 ± 14.98	29.49 ± 11.13	0.328
Femoral BMD (g/cm^2^)	1.04 ± 0.12	0.82 ± 0.06	0.68 ± 0.05	<0.001
Femoral T-score	0.04 ± 0.90	−1.66 ± 0.40	−2.79 ± 0.28	<0.001
Lumbar spine BMD (g/cm^2^)	1.22 ± 0.20	1.07 ± 0.14	1.05 ± 0.19	<0.001
Lumbar spine T-score	0.20 ± 1.58	−0.96 ± 1.18	−1.21 ± 1.57	<0.001

Continuous variables are expressed as mean ± SD. Only participants with available femoral BMD measurements were included in this analysis (*n* = 188).

**Table 4:** Correlation analysis of serum parathyroid hormone concentrations with bone mineral density and selected clinical and biochemical variables.

Correlated Variables	R	*p*-Value
PTH—Lumbar spine BMD (g/cm^2^)	−0.12	0.37
PTH—Lumbar spine T-score	−0.11	0.37
PTH—Femoral BMD (g/cm^2^)	−0.36	<0.001
PTH—Femoral T-score	−0.35	<0.001
PTH vs. FEP	0.29	<0.001
Age vs. eGFR	−0.40	<0.001

## Data Availability

The data presented in this study are available on request from the corresponding author. The data are not publicly available due to privacy restrictions.

## Abbreviations

The following abbreviations are used in this manuscript:

BMD	Bone Mineral Density
CaSR	Calcium-Sensing Receptor
CKD	Chronic Kidney Disease
CKD-EPI	Chronic Kidney Disease Epidemiology Collaboration
FEP	Fractional Excretion of Phosphate
FGF23	Fibroblast Growth Factor 23
eGFR	Estimated Glomerular Filtration Rate
HbA1c	Glycated Hemoglobin
IRMA	Immunoradiometric Assay
PTH	Parathyroid Hormone
VDR	Vitamin D Receptor
